# Perceptions of older people regarding drone-delivered defibrillators for out-of-hospital cardiac arrest: a qualitative study

**DOI:** 10.29045/14784726.2025.6.10.1.10

**Published:** 2025-06-01

**Authors:** Owen Finney, Kate Snowdon, Adonia Mckellow, Kayleigh Geen, Chris Wilkinson, Graham McClelland

**Affiliations:** University of Sunderland; North East Ambulance Service NHS Foundation Trust ORCID iD: https://orcid.org/0000-0003-3744-2710; University of Sunderland; North East Ambulance Service NHS Foundation Trust; University of Sunderland; University of Sunderland; University of York; South Tees NHS Foundation Trust ORCID iD: https://orcid.org/0000-0003-0748-0150; North East Ambulance Service NHS Foundation Trust; Northumbria University; Newcastle University; University of Hertfordshire ORCID iD: https://orcid.org/0000-0002-4502-5821

**Keywords:** AED, ambulance, bystander, drone, paramedic

## Abstract

**Introduction:**

Out-of-hospital cardiac arrest (OHCA) presents a significant public health challenge. Bystander utilisation of automated external defibrillators (AEDs) can improve survival. Drone delivery of AEDs may improve rates of bystander defibrillation. However, whereas most cardiac arrests occur in older people, there is minimal evidence on the perceptions of older people regarding AED delivery by drone. The aim of this study was to explore the perspectives of individuals aged 65 years and over on the use of drone technology for AED delivery in OHCA situations.

**Methods:**

Semi-structured qualitative interviews were undertaken to gather insights into participants’ perceptions about drone AED delivery. Responses were thematically analysed.

**Results:**

Three main themes were identified from 12 interviews conducted between May and July 2024: (1) the interaction between the human and the drone; (2) perceived societal benefits of drone AED delivery for OHCA; and (3) safety and public perception. Participants expressed complex reactions to drone-delivered AEDs, and expressed concerns about correct AED usage and the emotional difficulty of leaving a patient unattended. Many anticipated guilt about possibly being unable to use the AED effectively in high-stress situations. Participants acknowledged the potential for drones to save lives by reducing response times in OHCA, but raised concerns about safety and public education. There was a strong consensus on the importance of public education and training to build confidence in using both AEDs and drone technology.

**Conclusion:**

Although participants appreciated the rapid delivery of AEDs via drones for OHCA, they expressed significant concerns about their own ability to use the AED alongside the emotional burden associated with emergency situations. The findings emphasise the need for enhanced public education and psychological support to ensure effective bystander intervention in general. Additionally, prior to any roll-out of drone-delivered AEDs, there should be a specific programme of education to bridge the gap between technological acceptance and practical application.

## Introduction

Out-of-hospital cardiac arrest (OHCA) is a significant public health issue in the UK, with approximately 30,000 cases receiving pre-hospital resuscitation annually by NHS ambulance services across the four nations (Drennan et al., 2021). Survival rates from OHCA remain low in the UK, with around 9% survival at 30 days (Out-of-Hospital Cardiac Arrest Outcomes (OHCAO), 2023). Early defibrillation is an important determinant of survival (Berg et al., 2020; Nolan et al., 2006).

Although the use of automated external defibrillators (AEDs) by members of the public is associated with improved survival in OHCA (Perkins et al., 2021; Smith et al., 2017; Torney et al., 2020), they are used in fewer than one in 10 OHCAs (Perkins et al., 2021). Factors contributing to this low usage include lack of knowledge, awareness, willingness and accessibility, among other things (Burgoine et al., 2024; Smith et al., 2017).

The feasibility of drone delivery of an AED has been demonstrated (Cheskes et al., 2020; Fischer et al., 2023; Schierbeck et al., 2023), although there is limited data on the potential impact on clinical outcomes. Furthermore, studies on the human factors associated with drone-delivered AEDs have tended to include younger, more physically capable participants or trained professionals, with limited exploration of older adults’ perspectives (Baumgarten et al., 2021; Zègre-Hemsey et al., 2020). This is a significant limitation, as OHCA predominantly affects individuals over 64 years of age (Perkins et al., 2021).

The research team aimed to explore the thoughts, feelings and opinions of older people regarding the use of a drone-delivered AED in OHCA.

## Methods

### Qualitative approach and research paradigm

A generic qualitative research design was employed, as per that summarised by Cooper and Endacott (2007), that was based upon the literature from Caelli et al. (2003) and Merriam (1988). The research was conducted using semi-structured interviews with a topic guide to explore participants’ perceptions. This article has been reported as per the Standards for Reporting Qualitative Research (SRQR) guidance (O’Brien et al., 2014).

### Research question

What are the thoughts, feelings and opinions of older people regarding the use of a drone-delivered AED in OHCA?

### Researcher characteristics and reflexivity

The principal researchers who conducted the interviews and data analysis come from a constructivist background, which shaped the study’s interpretative approach. Both researchers engaged in regular reflexive practices, such as journaling and peer debriefing, to critically evaluate their personal perspectives and their potential influence on data interpretation. Their professional training as paramedics informed their understanding of the research topic, providing valuable insight into participants’ experiences but also necessitating ongoing reflexivity to ensure that preconceptions did not unduly shape the analysis. Efforts to address this included maintaining a reflexive diary, discussing analytical decisions within the research team and explicitly acknowledging their positionality in shaping the research process and findings.

### Context

The study was conducted at the University of Sunderland (UoS), with participants recruited from the UoS Patient Carer Public Involvement (PCPI) programme. Interviews were conducted either face to face or via Microsoft Teams, depending on participant preference.

### Sampling strategy

Participants were purposively sampled using a gatekeeper from the PCPI programme to ensure a diverse representation of backgrounds, including age, sex and previous experience of cardiopulmonary resuscitation (CPR). The gatekeeper approached all potential participants from the PCPI programme who met the inclusion criteria of the study via email. In total, 12 members of the PCPI programme agreed to take part in the research after email contact. Our sample of 12 participants was chosen for pragmatic reasons rather than to achieve data saturation. Following Braun and Clarke’s critique, we acknowledge that the concept of data saturation, as information redundancy, is not consistent with the principles of reflexive thematic analysis, where meaning is shaped through interpretation rather than being simply uncovered from the data (Braun & Clarke, 2019).

### Inclusion and exclusion criteria

Participants were eligible if they were aged 65 years or over and were a member of the PCPI programme at UoS. Currently registered healthcare professionals were excluded.

### Ethical considerations

Ethical approval was obtained from the UoS university ethics review process (Reference number: 024260). Participants provided written informed consent prior to taking part, with the option to withdraw from the study at any time without providing a reason. Participant characteristics are reported in an aggregated fashion, and all direct quotes are anonymised to preserve confidentiality. Data were securely stored in accordance with institutional guidelines, with access restricted to the research team to ensure privacy. Care was taken to phrase interview questions sensitively, recognising the potential for participants to share personal or emotive experiences. To support participant well-being, follow-up email check-ins were conducted by a gatekeeper unaffiliated with the research team, providing an opportunity for participants to raise any concerns or access support if needed.

### Data collection

Data were collected between May and July 2024. The interviews featured seven open questions with a training video provided by researchers at Warwick University to give a visual demonstration of the technology. The interviews were audio recorded and subsequently transcribed verbatim. Demographic data were collected at the start of each interview, including age, sex, occupational status, ethnicity, self-reported disability, self-reported experience with technology and previous experience with delivering or being trained in CPR. Member checking was employed to validate the accuracy of the transcriptions; this involved sending back the completed transcripts to the participants to ensure that they represented the perceptions of the participants prior to data analysis.

### Data analysis

Thematic analysis was performed using an inductive and semantic approach (Braun and Clarke, 2021). The six-stage analysis process included familiarisation, coding, theme generation, theme review, theme definition and writing up. The coding process was performed individually by both principal researchers before working collaboratively. The analysis from the theme generation stage was then conducted collaboratively by the principal researchers. The quotes that were used were selected to illustrate the themes.

### Techniques to enhance trustworthiness

Trustworthiness was ensured through collaborative data analysis sessions, member checking and the use of a systematic thematic analysis process.

## Results

Interviews were conducted with participants between May and July 2024. In total, 12 participants (five male, seven female) were interviewed. Of these, 50% (6/12) reported previous experience of performing or being trained in CPR, and all reported aptitude with day-to-day technology (Table 1). The interviews lasted a mean time of 22 minutes (range 18‒26 minutes). Three main themes were identified from the data (Table 2).

**Table 1.** Participant characteristics.

Mean age (SD)	70.3 (±3.2)
Sex (%)	
Male	5/12 (41.7%)
Female	7/12 (58.3%)
Occupation status (%)	12/12 Casually employed (100%)
Ethnicity (%)	12/12 White British (100%)
Disability (%)	4/12 (33.3%)
Self-reported previous technological experience (%)	12/12 (100%)
Self-reported previous CPR experience (%)	6/12 (50%)

**Table 2.** Progression from codes to themes.

Participant quotes (raw data)	Codes	Themes
*It’s what I do with the defib next that concerns me.* (Participant 7)	Hesitation in AED use	Theme 1: the interaction between human and drone: a complex response
*It’s a big ask to leave a patient to go and retrieve the AED, especially when you’re trying to focus on keeping them alive with CPR.* (Participant 4)	Dilemma of leaving the patient	
*Some older people are technophobes ... the technology involved with the drone might be a bit scary.* (Participant 6)	Fear of / apprehension about technology	
*The access to a defibrillator within three or four minutes is a real, real positive benefit.* (Participant 3)	Faster access to AED / better treatment	Theme 2: perceived societal benefits of drone AED delivery for OHCA
*What a wonderful opportunity to save someone’s life.* (Participant 9)	Improved survival rates / better treatment	
*If the drone crashes or has a malfunction, it could pose a risk.* (Participant 11)	Concerns about drone safety	Theme 3: safety and public perception
*The public needs to be aware of how drones work to prevent panic or misunderstanding.* (Participant 6)	Need for public education	
*If we put it on the local news ... that would reassure the public.* (Participant 12)	Public reassurance through campaigns	

### Theme 1: the interaction between human and drone: a complex response

The potential use of drones to deliver AEDs evoked a spectrum of reactions. While some participants were comfortable with the drone itself, concerns primarily related to using the AED.

*For me, not the drone itself ... It’s just a messenger ... But if you were expecting me to do anything with the drone, like if it landed and I would have to do something to send it on its way again, I would worry ... But the fact that it just comes, delivers the defib and goes away doesn’t bother me one little bit. It’s what I do with the defib next that concerns me.* (Participant 7)

*I think the technology is fantastic ... but when it comes to applying the AED, I’d be worried I might do more harm than good.* (Participant 1)

*Some older people are technophobes ... the technology involved with the drone might be a bit scary. Without instruction, the AED might be a bit frightening. *(Participant 6)

The idea of leaving a patient in cardiac arrest to retrieve the AED from the drone also posed an emotional challenge, adding stress to an already intense scenario. This situation evoked feelings of guilt for some participants, as they feared that their inability to effectively use the equipment might result in failure to save a life.

*I think you could be in that dilemma of thinking at this moment in time ... I’m really frightened of leaving them, and then the patient dying, but also dying when you’re not with them.* (Participant 10)

*It’s a big ask to leave a patient to go and retrieve the AED, especially when you’re trying to focus on keeping them alive with CPR ... It depends a lot on timing and whether you have anyone else to help.* (Participant 4)

For those envisaging themselves alone in these situations, the sense of helplessness was heightened.

*If you knew it got sent, but you couldn’t go get it for whatever reason ... how that would make you feel knowing it was there, and you couldn’t use it.* (Participant 10)

### Theme 2: perceived societal benefits of drone AED delivery for OHCA

There was a generally positive reaction to the potential of drone-delivered AEDs to speed up response times in OHCA. Participants saw value in the technology, acknowledging that it could improve survival rates by delivering AEDs faster than current ambulance response systems allow.

*If you’re waiting for an ambulance these days, you could be waiting quite some time before you get professional help ... The access to a defibrillator within three or four minutes is a real, real positive benefit.* (Participant 3)

*I would think it was marvellous. If the device is delivered to me before the ambulance gets there, what a wonderful opportunity to save someone’s life.* (Participant 9)

The concept was viewed as innovative, with participants recognising that, in real-world scenarios, avoiding delay increases the chances of survival. However, their excitement was tempered by practical concerns about the drone’s ability to navigate real-world environments, such as busy streets or rural areas.

*I think it would be a distraction for people, passers-by or drivers.* (Participant 6)

*I often drive along Seaham [town in North East England] and there’s model aeroplanes, and sometimes they’re a distraction when you’re driving.* (Participant 4)

### Theme 3: safety and public perception

While participants saw value in drone technology, many raised concerns about its safety and the public’s response to seeing drones used in medical emergencies. Some worried that a malfunction or crash could pose risks to bystanders, especially in urban or congested areas.

*If the drone crashes or has a malfunction, it could pose a risk.* (Participant 11)

*My concern is the safety issues ... the drone should be very carefully sorted out.* (Participant 1)

These comments reflected a shared apprehension about the potential for technical failures and the consequences they might have in high-density environments.

The technology itself was also viewed as potentially intimidating or even frightening to the public, particularly for those unfamiliar with drones.

*Well, the only negatives I can think of is the actual drones, and the only mention of drones in the public at the moment are used for combat and war.* (Participant 4)

Such associations raised concerns that the public might misinterpret the presence of drones at emergency scenes, potentially causing undue alarm or hesitation.

*The public needs to be aware of how drones work to prevent panic or misunderstanding.* (Participant 6)

Participants noted that the public might need reassurance to prevent panic when they see drones in action, and that public education and training were crucial to the success of drone-delivered AEDs. They emphasised the importance of ensuring people know how to use the AED properly and understand how the drone system works to foster confidence and capability in emergency situations.

*It’s important to educate people about how to use the AED and what to expect when the drone arrives.* (Participant 3)

Suggestions ranged from offering awareness sessions on AED use to providing instructional videos or materials that could accompany the device.

*If we put it on the local news ... that would reassure the public. Most people might think, ‘It’s a drone, I haven’t seen those before’, but some might be alarmed.* (Participant 12)

Participants felt that such initiatives would significantly reduce hesitation and improve the public’s overall reaction to drones. They believed that educational campaigns could help inform people about what to expect when a drone arrived on the scene, thus reducing fear or anxiety.

## Discussion

There was a contrast between the participants’ reported comfort with drone delivery technology and their apprehension about using an AED. Although there was enthusiasm for drone-delivered AEDs, many were hesitant about their ability to use the AED itself. Previous work has reported that many participants accepted the drone technology as an efficient delivery tool, but their concerns arose from whether bystanders would have the knowledge and skills to use the AED once it arrived (Sedig et al., 2020). This reflects a critical barrier: technology acceptance does not necessarily equate to application readiness.

In one recent study, four in 10 of the general public reported familiarity with AEDs, but just one in 20 reported knowing how to use one (Huang et al., 2020). Although people may understand the purpose of AEDs, practical knowledge remains limited. This could reduce the overall effectiveness of drone-delivered AEDs in emergency situations, and suggests that improved population awareness of how to use an AED is necessary, regardless of the mode of delivery.

A recurring concern among participants in this study was the fear of misusing the AED and causing harm. Smith et al. (2017) observed that many people feel more comfortable waiting for someone skilled in AED use, rather than intervening themselves. This is reinforced by Gonzalez et al. (2015), who found that over 60% of their respondents were unaware that AEDs could be used by non-medical personnel. This fear of causing harm and a preference for deferring responsibility to professionals highlights the need for better public education and awareness around AED use (Hawkes et al., 2019; Huang et al., 2020). While the arrival of drones is welcomed as a positive development, the potential hesitation or anxiety in using AEDs could undermine the life-saving potential of this intervention.

### Emotional and psychological factors

In addition to technical challenges, emotional and psychological factors significantly affect how individuals respond to emergencies (Poranen et al., 2022). Many participants expressed concerns about leaving a patient to retrieve an AED, reflecting the emotional burden and decision-making complexity in high-stress situations. This conflict between the need for immediate defibrillation and the fear of abandoning a patient underscores the importance of addressing both the practical and psychological aspects of emergency responses.

Previous studies show that emotional stress can hinder effective action, including the retrieval and use of an AED (Fredman et al., 2016). This ‘mental locking’ may be alleviated by using simple instructions, such as focusing on continuous CPR supported by clear communication from an emergency call handler, which is likely to be more effective in such situations (Dalby-Pedersen et al., 2024). Where there is a sole bystander, concerns about leaving a person in cardiac arrest to retrieve an AED are well-founded, as AED retrieval (even from a short distance) is associated with a substantial interruption to CPR, which is likely to be associated with adverse neurological and survival outcomes (Finney et al., 2025).

Linking back to the technological dilemma, emotional stress often exacerbates concerns about personal capabilities. Farquharson et al. (2023) highlighted the perceived lack of capability as a significant barrier to bystander intervention, while Abelsson et al. (2020) found that training directly correlates with the willingness to perform CPR. When individuals self-evaluate under stress, they often underestimate their abilities, which can lead to inaction due to fear of negative consequences, as noted by Shams et al. (2016).

Participants in this study expressed similar fears about AED use, with many worried that their actions could cause harm. This aligns with the findings of Mathiesen et al. (2016), where bystanders reported significant self-criticism and anxiety after attempting to assist during a cardiac event. The most common barriers to AED use included fear of legal consequences and concerns about improper technique (Huang et al., 2020).

This emotional burden further explains why many respondents in this study and others (e.g. Gonzalez et al., 2015; Smith et al., 2017) feel AEDs should only be used by professionals. The ‘diffusion of responsibility’ effect (Darley & Latane, 1968), where the involvement of a medical professional legitimises bystander intervention, may reduce the anticipated fear of negative outcomes, making bystanders more willing to act if they feel they have permission or guidance from a qualified professional.

Last, feelings of guilt were expressed as a potential consequence of being unable to use the AED effectively, despite its delivery by drone. Post resuscitation, bystanders may experience a range of psychological sequelae, including self-blame and feelings of inadequacy, particularly if the outcome is poor (Brinkrolf et al., 2021; Mathiesen et al., 2016). Evidence also underscores the importance of debriefing lay responders after such incidents, as debriefs provide crucial coping mechanisms, help alleviate self-criticism and improve the responder’s confidence for future emergencies (Møller et al., 2014; Schnaubelt et al., 2023). Debriefing for bystanders may be an important factor in successfully implementing this technology to current ambulance response models.

### Public perception

Participants acknowledged the benefits of faster AED delivery through drones but expressed significant concerns around safety, noting that drones could seem intimidating or intrusive. This highlights the need for public education as part of any deployment strategy, aiming to build awareness around the purpose and operation of drones to mitigate fears and promote acceptance. Public endorsement of technology often depends on its perceived social benefit, as illustrated by Sedig et al. (2020), who noted increased acceptance when drones were viewed as life-saving tools. Additionally, 87% of respondents supported drone implementation when associated with search and rescue (PwC, 2019), highlighting the importance of framing such technology for maximum societal impact.

The broader public’s perception of drones is shaped heavily by media, hobbyist use and popular culture, where drones are sometimes depicted in extreme or unrealistic scenarios. For example, Kunze and Frommer (2021) suggest that mainstream representations, such as those in *The Fifth Element*, may skew public expectations of drones. Reddy and DeLaurentis (2016) found that although 93% of people are aware of drones, their understanding is mostly derived from movies or news, which can paint the technology as fantastical or far-fetched. Additionally, media portrayals of military drones foster associations with weaponry, privacy invasion and danger (Boucher, 2016), creating a fear-driven narrative. Commonly associated terms like ‘military’, ‘monitoring’ and ‘danger’ (Eißfeldt et al., 2020) reflect public anxieties that may affect willingness to engage with drone-delivered AEDs.

Concerns about potential drone malfunctions or accidents also persist, with participants worrying about risks to bystanders and the reliability of AED delivery. The literature echoes these anxieties, often citing drone misuse and accidents as primary issues (Miron et al., 2023; PwC, 2019). In a study by Truog et al. (2020), drone crashes in communities with limited public consultation sparked negative responses, especially when surveillance was involved. These cases illustrate the importance of transparent public engagement in gaining acceptance for AED drone delivery systems.

### Future research

It is crucial to maintain a continued emphasis on OHCA recognition, CPR and the use of AEDs among the public. Before implementing drone technology for AED delivery, comprehensive public education will be essential to ensure that individuals accept the technology. Given the identified feelings of guilt surrounding potential failure and the anxiety associated with leaving a patient unattended, more research is necessary to further explore these emotional factors and the ways in which they influence bystander interventions during drone AED delivery in OHCA. Last, although not directly related to the scope of this project, the team recognises that further research to better identify OHCA circumstances in which drone delivery is likely to be of net benefit in relation to interruption of CPR, which includes bystander factors, will be paramount for implementing this intervention.

### Limitations

This study is topical and explores an important and under-evaluated area. However, the authors acknowledge the limitations of this research. The participants were from a single-centre PCPI group, who may have above-average exposure to clinical environments compared to the general population. This could limit the generalisability of the findings, as experiences with medical technologies may vary across different groups.

Additionally, the study sample lacked ethnic diversity, which is a limitation in terms of capturing the full range of experiences and cultural perspectives on drone-delivered AEDs. This was not a targeted or intentional exclusion, but rather a reflection of the participant pool available at the time of data collection. Future research could benefit from including a more diverse range of participants to explore how cultural and socio-economic factors might shape attitudes towards emerging healthcare technologies.

Finally, the training video used in this study depicted a smooth, complication-free AED delivery, designed to illustrate the potential functionality of the drone-AED system in an ideal scenario. While this was effective in demonstrating the basic concept of the technology, it may have unintentionally shaped the participants’ perceptions of the process as more straightforward and error free than it would likely be in real-world situations. The absence of real-life, complication-laden footage may have contributed to an overly positive impression of the drone’s capabilities, potentially downplaying concerns about technical failures, user errors or other challenges that could arise in actual deployments. In future studies, incorporating more realistic scenarios, including the potential for complications, could help provide a more balanced view of the technology’s practical applications.

## Conclusion

This study explores the perceptions of older adults regarding the delivery of AEDs by drone in OHCA. While they generally embraced the speed and accessibility of drones in emergency scenarios, their confidence in using the AED itself was a significant concern to participants. Emotional factors played a substantial role, as participants expressed concerns about leaving a patient to retrieve the device, and articulated fears of misusing the equipment in a way that would cause harm.

These findings underscore the need for targeted public education on AED use, as well as psychological support to mitigate stress in high-pressure situations. Addressing these human factors is essential to enhance the effectiveness of drone-delivered AED systems and to ensure that bystanders are equipped and confident to act in emergencies.

## Acknowledgements

We are grateful to Dr Lesley Scott at the University of Sunderland for acting as a gatekeeper in this study. Thanks also to Dr Christopher Smith at Warwick University for providing the training video to facilitate this study.

## Author contributions

OF conceptualised the project. OF, GM, CW and KS designed the study. OF applied for ethical approval. OF and KS collected the data. AM and KG transcribed the data. OF and KS analysed the data and interpreted the results. OF, GM, CW and KS wrote the manuscript. All authors were involved in the review process and all authors approved the final draft. OF acts as the guarantor for this article.

## Conflict of interest

GM is on the editorial board of the *British Paramedic Journal*.

## Ethics

Ethical approval was obtained from the University of Sunderland ethics review process (Reference number: 024260). The study was conducted as per the protocol and ethical approval. No adverse events occurred from this research.

## Funding

None.
